# The relevance and application of measures of thermal tolerance to fish ecology, conservation and aquaculture

**DOI:** 10.1093/conphys/coag044

**Published:** 2026-07-07

**Authors:** Anthony K Gamperl, E Don Stevens

**Affiliations:** Department of Ocean Sciences, Memorial University of Newfoundland and Labrador, 0 Marine Lab Road, St. John’s NL A1C 5S7, Canada; Tropical Marine Ecophysiology Lab, Cape Eleuthera Institute, The Island School, Rock Sound, Eleuthera, The Bahamas; Department of Biomedical Science, Atlantic Veterinary College, University of Prince Edward Island, Charlottetown, PE C1A 4P3, Canada

**Keywords:** Adaptation, critical thermal maxima, critical thermal minima, hypoxia, incipient temperatures, incremental thermal maxima, lethal, physiology, sub-lethal, temperature

## Abstract

Over the past 20 years, the fish research community has invested considerable effort into measuring the thermal tolerance limits of many freshwater and marine species. We have learned a lot regarding mechanisms that determine tolerance limits, and how climate-related warming of aquatic environments may affect fish biology, ecology and distribution. However, in our opinion, some research has used methods that are likely to provide inaccurate estimates of thermal tolerance with respect to wild and cultured fish populations. Given the rate of global warming, the increase in the frequency and severity of heat waves, and that these challenges are often combined with other climate change-related stressors (e.g. hypoxia, increased pathogen prevalence), we need to ensure that future efforts are more effectual for species’ conservation and management. Herein, we provide a comprehensive and critical review of the methods currently used to measure the sub-lethal and lethal thermal limits of fishes, and discuss the relevance of these methods to fishes in the wild or in culture. In part, our aim is to educate fisheries managers and conservationists about what information is relevant to efforts to preserve species and biodiversity in the face of climate change. Finally, we provide explicit recommendations for researchers designing future thermal tolerance experiments.

## Background

Climate change has resulted in the annual average sea surface temperature between 60^o^ N and 60^o^ S (i.e. excluding polar regions) setting a record high of 20.87°C in 2024 (0.51°C above the 1991–2020 average; Copernicus Climate Change Service, 2024), and increases in lake surface and river temperatures in many regions of ~0.3–0.5°C per decade (Liu *et al.,* 2020; Woolway *et al.,* 2020). Such changes impact the survival and fitness of aquatic organisms including fishes, in part, because they also face increases in the frequency and severity of heat waves, hypoxic events, ocean acidification and habitat loss (Breitburg *et al.*, 2018; Frolicher *et al*., 2018; Laufkötter *et al.,* 2020; Woolway *et al.,* 2020; Barbarosso *et al*., 2021; Oliver *et al.,* 2021; Sampiano *et al.,* 2021, Smucker *et al.,* 2021; Wu 2002; Cheng *et al.,* 2023). In response to these challenges, there has been a marked increase in the number of publications on the thermal and other environmental limits of various fish species [e.g. see Bayat *et al.,* 2025; Raby *et al.,* 2025a].

There are two main reasons why we study fish thermal tolerance. First, to understand the cellular and physiological mechanisms that result in a loss of performance (e.g. metabolic capacity, growth, reproductive fitness, swimming ability) at suboptimal temperatures or temperature-dependent mortality. This research has led to a growing consensus that numerous mechanisms determine the thermal tolerance of fishes (e.g. see Ern *et al.,* 2023), and that a major research challenge is integrating information across levels of biological organization (McKenzie *et al.,* 2016; Cooke *et al.,* 2017a and 2017b; Illing & Rummer, 2017; Lefevre *et al.,* 2021, Cooke *et al.,* 2022; Ern *et al.,* 2023; McKenzie *et al.,* 2023; Seebacher *et al.,* 2023). The second reason is to provide accurate and relevant data on sub-lethal and lethal temperature tolerances that may be useful to conservationists, ecologists and fisheries and aquaculture managers in protecting fishes from the vagaries of climate change or to predict changes in their distribution. The latter is the focus of this perspective.

We argue that many studies on thermal tolerance have limited predictive value regarding a species’ vulnerability to climate change (i.e. do not provide accurate data on their thermal tolerances). This is due to many factors, including: (i) the species or life stage being studied, (ii) the experimental model and/or protocol used to measure thermal tolerance and (iii) that there are too few studies that simultaneously expose fish to situations or stressors that co-occur with sub- or supra-optimal temperatures in nature (i.e. activity, pathogens and hypoxia being the most important) or that examine temperature effects over a transgenerational time scale.

## Choice of species and life stage

For species/sub-species (for which we have information) that have gone extinct, or have been extirpated, the primary factors responsible for their disappearance has been landscape changes (habitat loss) or drought, not high temperatures. However, there have been notable localized fish mortality events related to climate change-induced heat waves (Genin *et al.,* 2020; Tietbohl *et al.* (2025)) and warm stream temperatures (Martins *et al*. 2011; Barnes (Katersky *et al.,* 2011). In addition, it is clear that many fishes are altering their latitudinal or depth distribution to avoid warming waters (Perry *et al.,* 2005; Dulvy *et al.,* 2008; Pinsky *et al.,* 2013; Pinsky *et al.,* 2020; Dahms & Killen, 2023). Thus, there is an urgent need to understand how climate-related changes in environmental conditions are affecting fish populations, what species could be at risk of disappearing from particular locations/regions, and the potential impacts on local economies and biodiversity. However, research on the thermal tolerance and climate change susceptibility of fishes has largely focused on a few species, mainly from temperate regions, and more research on Arctic and tropical species is sorely needed.

For a particular species, thermal tolerance depends on life stage (e.g. see Dahlke *et al.,* 2020). It is often a particular life stage that is potentially vulnerable to the effects of temperatures that approach or exceed its thermal limits. Thus, it may be of limited value to assess the critical thermal maximum (CT_Max_, a measure of acute thermal tolerance; see below) of other life stages if the data are to be relevant to climate change. For example, measuring the CT_Max_ of Atlantic salmon (*Salmo salar*) eggs, alevins or fry is of limited ecological relevance as this species spawns in the fall, and these life-history stages develop in cold/cool waters in the winter and spring. It is the stream resident parr, smolts and mature adults (i.e. those returning to spawn) that experience high summer temperatures, and these life-stages should be the focus of studies that aim to estimate this species’ vulnerability to large, short-term, increases in temperature. Instead, measuring the effects of prolonged exposure to temperatures a few degrees higher than current levels may be more relevant to assessing the potential impact of climate change on the salmon’s eggs, alevin and fry. Similarly, while Bluefin tuna (*Thunnus thynnus*) eggs are found in the top 20–25 m of the water column and can be exposed to very high summer temperatures (Moyano *et al.,* 2026), the adults are regularly found at depths between the surface and 500 m and are very adept at behavioural thermoregulation. Thus, for many species, it would be best if researchers were to conduct a risk (or vulnerability) assessment before prioritizing which life-stage to work on and the specific thermal tolerance protocol to be utilized.

Finally, the thermal tolerance of cultured or laboratory reared (i.e. domesticated) fish often differs from that of wild individuals (Vincent, 1960, Carline & Machung, 2001; Morgan *et al.,* 2019 and 2022; Debes *et al.,* 2021; Turko *et al.,* 2023; Thorarensen & Nelson, 2024), including that of the F1 generation from wild parents s (Morgan *et al.,* 2019). Thus, we recommend that researchers use wild caught fish whenever possible if the goal of the research is to assess the thermal tolerance of natural populations.

Clearly, selecting an appropriate species and identifying the life stage(s) that is/are at greatest risk to temperature changes, will allow for more robust projections on the consequences of climate for fish populations, as well as be the best guide for management and conservation efforts.

## Choice of protocol

There is an ever growing body of data on fish acute thermal tolerance, and this had led to several reviews that provide recommendations on how such studies should be performed (see Desforges *et al.*, 2023; Morash, 2024; Raby *et al.,* 2025a). However, many of these studies: (i) use protocols that have limited relevance to fishes in their natural or cultured environments, (ii) limit their investigations to single, simply measured, physiological metrics and (iii) assert that this information indicates how close current temperatures and conditions are to a species’ limits, and can be used to make recommendations with respect to their conservation and management. The latter point is a particularly concerning one. Many managers who are involved in setting fisheries and conservation policies and regulations often have limited knowledge of fish thermal biology and physiology, and thus, depend on the research community to provide them with relevant information.

A number of factors have contributed to this trend. However, this has resulted in the increasing use of protocols that can be completed quickly on a large number of fishes, and misconceptions about what is actually being measured. For example, few studies determine the time scale/temporal nature and magnitude of changes in temperature and environmental conditions to which a given species is exposed to in nature. Furthermore, even when they do or if the information is available, they are often not used in evaluations of thermal tolerance. Finally, many of the more in-depth approaches that look at even one or two aspects of physiological regulation related to environmental tolerances take considerable time and training and require expensive/technologically complicated equipment and control systems. Thus, such studies require substantial funding and training compared with those using respirometry on resting fish and ‘reduced’ cardiac preparations (see below). Our understanding of fish thermal tolerance will progress at a much greater pace, and benefit efforts to conserve species and biodiversity, if we heed the advice of Platt (Platt, 1964), and conduct better and more relevant experiments.

The above was the impetus for writing this perspective. Research on fish environmental physiology and tolerances has grown enormously over the past 15 to 20 years, and collectively, we have made tremendous progress in understanding the mechanisms that determine fish acute and long-term thermal tolerance and intra-specific and inter-specific differences (Ern *et al.,* 2016; Jutfelt *et al.,* 2018; Lefevre *et al.,* 2021; Ern *et al.,* 2023; Desforges *et al.*, 2023; Kuchenmüller *et al.,* 2024; Silva-Garay *et al.,* 2025). However, we need to step back and reflect on what is being accomplished, what species/taxa should be prioritized, and whether we have become too ‘method’ as opposed to ‘problem’ oriented. Below, we separate thermal tolerance into methods that examine the ability of fishes to tolerate and/or resist acute (over several hours to a few days) temperature changes vs. those that are long-term (i.e. over weeks, months or longer).

## Acute thermal tolerance

### Incipient lethal and critical thermal tests

Regarding the upper and lower tolerance of fishes to acute temperature change, the two most used protocols are the upper and lower incipient lethal temperature (UILT and LILT, respectively) (Fry *et al.,* 1942; Fry, 1947) and the critical thermal maximum and minimum (CT_Max_ and CT_Min_) tests (Becker & Genoway, 1979; Desforges *et al.*, 2023). The two former tests were extensively used in the past, and provided seminal information on the thermal niches of various fishes, how thermal history (acclimation) influences temperature tolerance, and several other aspects of fish thermal biology (see Fry & Gibson, 1953; Brett, 1956). These tests involve acclimating fish to a particular temperature and then recording the temperature at which a proportion of the fish can survive for a defined period after being abruptly exposed to a new temperature (typically 50% for 96 h). However, this protocol has questionable ecological relevance. It takes more than a few days, and up to 3–4 weeks, for the thermal-dependent physiology of fishes to completely adjust after an abrupt change in temperature; i.e. for the process of acclimation to be completed (Doudoroff, 1942; De Bonville *et al.,* 2025; Raby *et al.,* 2025a). Even in the few cases where such abrupt changes in temperature occur in nature (e.g. when aquatic organisms cross a thermocline or enter the discharge water from power plants; Verones *et al.,* 2010; Elder and Seibel, 2015), these temperature changes rarely expose fish to lethal or near lethal limits. Furthermore, the use of death as an experimental endpoint should be avoided whenever possible. Thus, we recommend that their use be discouraged, and that regulatory guidelines for environmental temperatures be re-written so that they do not rely on such metrics.

Use of the CT_Max_ and CT_Min_ tests, where temperature is increased or decreased at a constant rate until resting fish lose equilibrium or show other major signs of system level dysfunction, has grown tremendously over the years, and studies using this protocol have provided extremely valuable information on the acute thermal tolerance of many fish species and their underlying physiological mechanisms. Recently, several authors have proposed ‘best practices’ so that CT_Max_ (and CT_Min_) values between species and life-stages can be compared (Desforges *et al.*, 2023; Ern *et al.,* 2023; Raby *et al.,* 2025a). However, the applicability of CT_Max_ and CT_Min_ methodology to fish ecology and conservation should be questioned for many fish species. This protocol involves increasing temperatures over several hours and by a large amount (~5 to 10°C+ depending on acclimation temperature; see below) until the fish loses equilibrium. The loss of equilibrium signifies a failure in the fish’s ability to control its movement and maintain an upright position in the water column, a condition that would lead to death in nature as the fish can no longer escape from the stressful conditions or predators. However, changes in temperatures of this magnitude and timeframe, and that approach or exceed a fish’s upper thermal tolerance, occur in only a few aquatic environments. They do occur in tidepools, salt marshes, mangrove creeks, coastal rivers in summer and those that experience periods of low flow and/or are in hot arid regions, in shallow pools or ponds, and in urban areas following heavy downpours (see Table 1). However, for most fishes, this protocol does not represent what they experience in nature. In most aquatic environments (including, pelagic marine environments, coral reefs and large lakes), fish are more likely to be challenged by seasonal temperature highs and lows that approximate their upper or lower thermal limits, or temperature increases of a few degrees lasting multiple days or a few weeks (i.e. changes characteristic of heat waves and cold snaps). Thus, protocols such as the incremental thermal maximum or minimum tests (IT_Max_ and IT_Min_, respectively; Zanuzzo *et al.,* 2019; Vadboncoeur *et al.*, 2023), the ITD_Max_ test (Iacarella *et al.,* 2025a), or the protocol recently used by McMahon *et al.* (2024), are more suitable for examining aspects of their thermal tolerance and biology that have ecological implications. In the latter study, to examine the potential effects of marine heat waves on the coral reef fish *Lutjanus carponotatus,* water temperature was maintained for 4 weeks at 1 and 2°C above normal maximum sea surface temperatures. This is because a marine heat wave is defined as episodes of warmer water lasting longer than 5 days (Hobday *et al.,* 2018), and MHWs have been shown to last ∼5–30 days at sites on the Great Barrier Reef (AIMS, 2017).

**Table 1:** Data collected in various environments where diurnal fluctuations in temperature are considerable, including the maximum and minimum temperatures and rate of heating

**Habitat**	**Location**	**Minimum Temp (°C)**	**Maximum Temp. (°C)**	^**o**^ **C h** ^**−1**^	^**o**^ **C min** ^**−1**^	**Reference**
Little Southwest Miramichi River	New Brunswick, CA	25.1	30.6	1.07	0.017	Caissie *et al.* (2012)
12 Mile Creek	Idaho, USA	13.3	19.1	1.83	0.031	Rodnick *et al.* (2004)
Bridge Creek	Idaho, USA	12.5	26.8	0.90	0.015	Rodnick *et al.* (2004)
Various Streams	British Columbia, CA	N/A	25.9–27.5	0.3–0.4	~0.006	Iacarella *et al.* (2025)
High Tidepool	Bamfield, CA	13.1	24	1.14	0.019	Richards (2011)
Freshwater Pool	California, USA	18.2	29	2.88	0.048	Desforges *et al.* (2023)
Rock Pool	Munster, SA	21	32.5	1.0	0.0017	Smit and Glassom (2017)
Mangrove Creek	Eleuthera, BH	29.2	39.2	1.15	0.019	Schneider *et al.* (2023)
Patch Reef	Eleuthera, BH	30.7	32.9	0.27	0.0045	Porter and Gamperl (unpubl.)
High Tidepool	California, USA	15.0	23.6	4.0	0.066	Fangue *et al.* (2011); Barnes (Katersky *et al.* (2011)
Salt Marsh	California, USA	15.0	33.4	2.68	0.044	Kraskura *et al.* (2024)
Artificial Pond	Manaus, BR	~ 29.0	35.0	~ 1.0	0.0017	MacCormack *et al.* (2003)
Beaufort Sea	Alaska, USA	4.2	10.1	3.4	0.0056	Gatt *et al.* (2019)
Urban	California, USA	20.8	25.6	N/A	~ 0.48	Somers *et al.* (2013)
Aquaculture Cage-Site	Nova Scotia, CA	5.0	15.0	0.2	0.003	Gollock *et al.* (2006)

Abbreviations: CA = Canada; BH = Bahamas; BR = Brasil; SA = South Africa; USA = United States of America. Note: data in the second to last row for the `urban' site are for stream temperatures due to runoff from hot paved areas following a rain storm. This temperature increase was over a very short duration; ~10–15 min.

Furthermore, we are concerned about several aspects of the recent review by Raby *et al.* (2025a) and how they may give readers a false impression about the utility of CT_Max_ and CT_Min_ tests that use very fast heating rates. Raby *et al.* (2025a) strongly advocate that a heating rate of 0.3°C min^−1^ (18°C h^−1^) be used in CT_Max_ experiments, in part because ‘rock pools and freshwater pools undergo extreme changes over a timescale of minutes’ (citing Smit & Glassom, 2017 and Desforges *et al.*, 2023). Furthermore, they state that warming rates from 1 to 18°C h^−1^ ‘produce no differences in CT_Max_’ (citing Elliott & Elliott, 1995) and indicate that the rate of warming needs to be fast enough to avoid ‘unwanted’ (partial) acclimation to intermediate temperatures as the temperature is increased or decreased’. The first statement is inaccurate. First, the fastest temperature increase reported in Desforges *et al.* (2023) was 0.048°C min^−1^ (~ 2.9°C h^−1^), and in Smit and Glassom (2017; we have analysed their raw data) was 0.017°C min^−1^ (~ 1.0°C h^−1^). Thus, in these two cited studies, it would have taken between 20 min and 1 h for a 1°C increase in temperature. Furthermore, we calculated acute temperature changes in several other marine and freshwater environments from published papers. With the exception of one paper that reported an increase in stream temperature of ~4°C in 10 min (Somers *et al.,* 2013) due to runoff from paved urban areas following a summer rain storm, acute increases in temperature ranged from ~0.3 to 4.0°C h^−1^ (see Table 1). Finally, using a uniform rate of 0.3°C min^−1^ (18°C h^−1^) or even 10°C h^−1^ can overestimate thermal limits for even small fishes because changes in body temperature lag behind that of the water (Fangue *et al.,* 2011; Jutfelt *et al.,* 2019; Sandrelli & Gamperl, 2023) Thus, with rare exceptions, 18°C h^−1^ is clearly much too fast and not representative of what fishes are exposed to in nature or in an aquaculture setting.

Second, Elliott and Elliott (1995) state that ‘critical thermal maximum increased asymptomatically with the rate of temperature increase’. For example, Table 1 of that paper shows that the CT_Max_ of Atlantic salmon heated at 18^o^ C h^−1^ was ~4.5°C higher than when this species was exposed to an ecologically relevant rate of heating (0.5°C h^−1^). This finding is consistent with the meta-analysis of Kingsolver and Umbanhowar (2018) who report a similar relationship. Specifically, that CT_Max_ increases by ~3.5°C between heating rates of ~1.0 and 18°C hr^−1^. In addition, while CT_Max_ is, in general, higher at fast vs. slow heating rates, it is not possible to accurately estimate what a fish’s CT_Max_ would be if heated at ecologically relevant rates as the relationship (difference) between CT_Max_ values obtained using high heating rates vary with species and life history stage. For example, even though CT_Max_ values for 22°C- acclimated zebrafish (*Danio rerio*) warmed at 18 and 2°C h^−1^ were significantly correlated (Åsheim *et al.,* 2020), this relationship had a very low *r*^2^ value (0.15). There was no such relationship in 34°C acclimated fish. Finally, which test resulted in the highest measure of thermal tolerance depended on acclimation temperature (i.e. they varied in both magnitude and direction). Moyano *et al.* (2017) showed that *CT_Max_* increased significantly in larval herring (*Clupea harengus)* at faster rates of warming, whereas no effect was observed in seabass (*Dicentrarchus labrax).*  Raby *et al.* (2025b) reported that 5 of the 6 species tested at warming rates of 18°C h^*−*1^ and 1°C h^*−*1^ had higher CT_Max_ values using the former rate that ranged from ~0.4 to 1.2°C, whereas the sand goby’s *(Pomatoschistus minutus)* CT_Max_ was lower by 1°C*.* Finally, De Bonville *et al.* (2025) showed that thermal acclimation dynamics differ considerably across fish species, and that in some species an increase of 1°C in CT_Max_ can be realized following even brief (i.e. 3 h) exposure to elevated temperatures. In summary, our analysis indicates that very rapid temperature increases do not predict ecologically relevant temperature limits, in agreement with a number of other authors (Rezende *et al.,* 2014; Bates & Morley, 2020; Iacarella *et al.,* 2025b).

The issue of the effect of heating rates on CT_Max_ values has recently been addressed by Jørgensen *et al.* (2021) and Ørsted *et al.* (2022). These authors propose that thermal death time (TDT) provides a mathematical foundation to model CT_Max_ as it considers both the intensity and duration of thermal stress. Their model applies to temperatures above permissive values (i.e. those that normally relate to biological metrics such as growth, activity and standard metabolic rate), where TDT depends on a balance between disruptive and regenerative biological processes that ultimately define a critical boundary temperature. This model has promise. However additional validation is required with regard to using TDT as a means for comparing CT_Max_ values between studies that use different heating rates. Furthermore, for some species estimates of TDT will result in significant errors with respect to their thermal tolerance under ecologically relevant conditions (Raby *et al.,* 2025a). Nonetheless, such an approach was recently utilized by Molina *et al.* (2024) to examine the relative contribution of plasticity and evolution to the heat tolerance of fishes.

The purported value of heating at very fast rates (i.e. 18°C h^−1^) is that it allows for an examination of fish thermal tolerance based on the basal/inherent physiological characteristics of an animal pre-exposed to a set of conditions. However, the recommendation that ‘the rate of warming needs to be fast enough to avoid unwanted (partial) acclimation to intermediate temperatures as temperature is increased or decreased’ (Raby *et al.,* 2025a) is not relevant in an ecological context. It disregards all compensatory processes that occur during exposure to warming or cooling temperatures. Fishes show considerable physiological plasticity when exposed to acute changes in temperature at realistic rates (see Table 1), and this greatly influences their thermal tolerance (see De Bonville *et al.,* 2025; Vallin *et al.,* 2025). As Desforges *et al.* (2023) effectively articulate, CT_Max_ can only effectively predict the ecological impacts of environmental warming if parameters influencing thermal limits, such as pre-exposure temperature and the rate of warming, are carefully considered.

Another major goal of research on thermal tolerance should be to identify factors at various levels of biological organization (i.e. from behaviour to genomic/genetic changes) that determine fish sub-lethal limits as these will impact important life history traits such as growth, reproductive fitness, etc. This is because these impacts are likely to have a much greater influence on most fish populations as compared with temperatures that exceed their upper or lower thermal limits. Furthermore, it is recognized that changes in transcript expression are the mechanistic link between genetic and cellular responses that determine organismal physiology and tolerance (Rivera *et al.,* 2021; Earhart *et al.,* 2023; Metzger *et al.,* 2024). However, critical thermal tests that use rapid rates of heating also have major drawbacks. First, levels (transcript or protein) of heat shock or other stress-related markers measured at a warming rate of 18°C h^−1^ are not quantitatively similar to those in organisms exposed to more ecologically relevant rates of heating. For example, Penman *et al.* (2023) showed that *hsp70*, *hsp90a* and *hsp90b* mRNA expression in the gill of white sturgeon (*Acipenser transmontanus*) was much lower in fish given a CT_Max_ test with a heating rate of 18 vs. 1.8°C h^−1^. These data strongly suggest that the magnitude of the heat shock response, and potentially the protection afforded by these key elements of the cell’s stress defence, will be underestimated if assessed using very fast vs. ecologically relevant rates of warming. Another major disadvantage of using very fast heating rates is that measurements of gene (transcript) expression at a particular sampling temperature will likely reflect transcription levels at a temperature several degrees cooler. This is because it can take considerable time before increases in transcript (mRNA) expression can be detected, and even longer for changes in protein levels to occur. Unfortunately, there is very limited data in this area. However, it has been reported that it takes at least 15 min at high temperatures, and longer as pre-test temperature is decreased, before changes in the transcript expression of the highly conserved heat shock protein 70 (*hsp*70) can be detected in the goby *Gillichthys mirabilis* (Buckley & Hofmann, 2004). Thus, at a starting temperature of 30°C and a heating rate of 18°C h^−1^, at any sampling point one would be measuring levels of transcript expression that occurred at a temperature ~4.5°C lower than when the samples were taken. This simple analysis agrees with Rivera *et al.* (2021). These authors emphasized that ‘timing is everything’ with respect to the interpretation of post-stress transcriptomic levels/patterns. In addition, they indicated that a true measure of cellular processes at the fish’s thermal limits (e.g. at what temperature stress is evoked?) is never obtained at very high heating rates, and that at best a temperature adjustment related to the rate of heating must be applied when using such biomarkers to predict when fish first experience sub-lethal stress. But how can this be done accurately given the very limited data on the effect of the rate of heating on the temperature-dependent induction of key transcripts? These shortcomings are clearly another major disadvantage of using rapid temperature changes in CT_Max_ and CT_Min_ tests as compared with ecologically relevant experimental paradigms where heating rates are <4°C h^−1^. Collectively, the above brings into question the relevance of CT_Max_/CT_Min_ tests for large numbers of fish species, and more specifically the use of heating rates that are extremely fast (e.g. 0.3°C min^−1^, 18°C h^−1^).

Using rates of temperature change that reflect those experienced by the fish needs to be the standard we apply to this metric of thermal tolerance if it is to be used to predict distribution, and inform conservation and management policies. Using CT_Max_ data based on experiments conducted at 18°C h^−1^ will, with rare exceptions, significantly overestimate the acute upper thermal tolerances of fishes, and could lead to standards and/or policies that do not protect, and at worst, endanger wild fish populations. While we agree that comparisons between species, life-stages and populations require that a standard heating rate be used, we strongly advocate for the use of 2°C h^−1^ as opposed to 18°C h^−1^. This value is in the middle of the range of recorded heating rates in nature (see Table 1) and has already been used in a large number of studies. Thus, there is a growing data base to which new information can be compared.

### The ‘rapid screening’ protocol/F_H,Max_ test

Casselman *et al.* (2012) introduced a ‘Rapid Screening Protocol’ and used it to examine whether the temperature that cardiac arrhythmias occur estimates the optimum temperature for aerobic scope for a particular species. This protocol involves anaesthetizing fish at their acclimation temperature, blocking cardiac cholinergic tone with atropine and maximally stimulating the heart with the adrenergic antagonist isoproterenol, and then warming this preparation at 10°C h^−1^ until the heart goes arrhythmic. This protocol has now been used in more than 40 studies to examine the effects of acute increases in temperature on fish cardiac performance limits. This is, in part, because of this protocol’s ‘high throughput’ capacity, and that measurements of F_H,Max_ offer functional insights into acute upper thermal limits (Ferreira *et al.,* 2014; van derWalt *et al*., 2021; Gilbert *et al.,* 2024).

However, it remains unclear as to whether these rate transition temperatures are accurate predictors of acute thermal limits (i.e. CT_Max_), or relevant to climate change. For example, only one study has directly compared the T_peak_ (which is purported to be the most appropriate measure of cardiac thermal tolerance; Gilbert *et al.,* 2024) value in fish free-swimming with those obtained using the F_H,Max_ test. Nonetheless, Sandrelli and Gamperl (2023) showed that Atlantic salmon allowed to recover for one month after heart rate biologger (Star-Oddi; Garðabær, Iceland) implantation had a *T*_peak_ value during a CT_Max_ test at 2°C h^−1^ that was more than 6°C higher than when measured using the using the F_H,Max_ protocol (and 7.8°C lower than the salmon’s CT_Max_). Furthermore, analysis of the supplemental data in Gilbert *et al.* (2024), when limited to studies that measured both CT_Max_ and *T*_peak_ and/or *T*_arr_, reveals two things. First, the latter two parameters differ from species’ specific values of CT_Max_ by ~5.6°C (mean 27.35%, range ~ 3–9°C) and 3.1°C (mean 13.4%, range ~ −1.4–8.7°C), respectively. Second, that although there were strong significant relationships between *T*_peak_ and CT_Max_ and between *T*_arr_ and CT_Max_ for these species, CT_Max_ values estimated from these relationships varied from ~ −3.8 to +3.3°C and ~ −5.2 to +4.6°C compared with measured CT_Max_ values, respectively (see Supplemental file). This degree of inter-study and inter-specific variability makes it extremely difficult to accurately estimate the acute upper thermal tolerance (CT_Max_) of any fish species based on metrics of cardiac thermal tolerance measured using the ‘Rapid Screening Protocol’/F_H,Max_ test.

In addition, its use is highly questionable in situations where fish are simultaneously exposed to other challenges, such as hypoxia prior to or during warming (see Schwieterman *et al.,* 2023) or when fish are swimming and warmed. In the F_H,Max_ protocol, a high dose of atropine is used to block cholinergic control of the heart, and Sandrelli and Gamperl (2023) recently showed that Atlantic salmon *in vivo* thermal tolerance is greatly reduced following atropine administration because the pharmacologically induced increase in F_H_ (tachycardia) results in a more rapid decrease in stroke volume (S_V_, the amount of blood pumped per heart beat) and premature cardiac failure. That said, cholinergic tone varies greatly between fish species (see Table 5 in Farrell & Smith, 2017), and thus, there may be a few species where this is not as great a concern. Furthermore, it is not possible to examine the influences of adrenergically mediated mechanisms on fish cardiac function and thermal tolerance alone, or in combination with hypoxia. In the ‘Rapid Screening Protocol’, fish are given a maximal dose of the adrenergic agonist isoproterenol before increases in temperature begin, and this precludes any meaningful data from being obtained with regards to the role of this critical support system and how it is affected by these two important environmental challenges. Finally, it does not appear that this protocol actually measures the maximum F_H_ of fishes. *In vivo* maximum F_H_ is ~40 beats min^−1^ (26%) higher in schoolmaster snapper (*Lutjanus apodus*) given a CTS_Max_ test (see below) vs. a CT_Max_ test (Durnford *et al.,* 2026), and thus, one should not assume that measures of *T*_peak_ and *T*_arr_ using the ‘Rapid Screening Protocol’ are accurate estimates of those in swimming fish when exposed to supra-optimal temperatures; or in fact, the maximum F_H_ that a given species can achieve.

In summary, while the F_H,Max_ protocol provides valuable information on fish temperature-dependent cardiac function, its plasticity, and the capacity of fishes to survive further climate-related increases in water temperature (see Strowbridge *et al.,* 2025), it should not be used as a proxy for a fish’s maximum acute thermal tolerance (CT_Max_). Values obtained for *T*_peak_ and *T*_arr_ are often well below a species’ CT_Max_ (i.e. beyond what would be considered ‘conservative’), and highly variable.

Alternative approaches to examining the effect of temperature on F_H,Max_ in free-swimming fishes are measuring bioelectric potentials (electrocardiograms) in freshwater fish using external electrodes (Altimiras & Larsen, 2000) or using implantable biologgers in larger freshwater and marine fish (i.e. > ~120–150 g currently) following complete recovery (see Sandrelli & Gamperl, 2023). The latter can be performed on many fish at the same time (and thus can be considered ‘high throughput’), and biologgers can be reused many times for such measurements. Furthermore, in these tests, both the fish’s F_H_ and thermal tolerance (i.e. CT_Max_, IT_Max_ etc.) can be measured, and this provides the opportunity to directly assess the relationship between these two metrics. Nonetheless, in situations where thermal stress is combined with other challenges such as exercise or hypoxia, F_H_ may not be the only parameter determining a fish’s cardiac output (Q̇); i.e. S_V_ can play a significant role (Farrell & Smith, 2017). Thus, Q̇, not, F_H_ should be measured if possible. Cardiac output can be directly measured by implanting Doppler® or Transonic® flow probes around the fish’s ventral aorta, placing the fish in either a chamber or swim tunnel, and connecting the flow probe leads to a flow meter and associated equipment. However, surgery and confinement themselves can affect a fish’s thermal tolerance and scope for cardiac function (Petersen & Gamperl, 2011; Sandrelli & Gamperl, 2023), and thus, the best experimental approach would be to measure Q̇ (and thus also F_H_ and S_V_) in free-swimming fish that have fully recovered from surgery. This was recently possible as Transonic Systems (Ithaca, NY, USA) successfully developed and marketed, a fully implantable, multivariate, bio-logger system that had the capacity to simultaneously measure blood flow, electrocardiograms (ECGs), swimming activity (as 3D acceleration) and body temperature in freely swimming fish (see Brijs *et al.,* 2019). Unfortunately, this product was discontinued in 2023.

### Critical thermal maximum while swimming

The above protocols are typically performed on resting fish. However, many fish species swim continuously or for prolonged periods, and thermal tolerance while swimming is likely to be different than at rest as the metabolic costs associated with swimming divert resources away from functions that support temperature-dependent changes in cellular processes or organismal physiology. To address this, Blasco *et al.* (2020) developed a critical thermal maximum while swimming (CTS_Max_) test, where fish swim at speeds of ~80–85% of their critical swimming speed (i.e. where oxygen consumption is near maximum, but anaerobic metabolism has not yet been recruited) while warmed at a constant rate until fatigue. To date, this protocol has been performed on only four species: pacu (*Piaractus mesopotamicus*) and tilapia (*Oreochromis niloticus*), Blasco *et al.* (2020, 2022); European sea bass, Nati *et al.,* 2023; and schoolmaster snapper, Nati *et al*. (2024) at a heating rate of 2°C h^−1^. Interestingly, these studies report that CTS_Max_ was ∼1.5°C less than the CT_Max_ for the tropical fish species, whereas in the temperate sea bass the difference was 4°C. Collectively, these data suggest that if fishes are exposed to an acute warming episode, the capacity to perform maximum (or near maximum) aerobic metabolic work for prolonged periods (i.e. hours) becomes limited at temperatures below those at which their survival is threatened. The above information is consistent with what would be predicated based on the relationship between aerobic scope and acute increases in temperature (see Steinhausen *et al.,* 2008), and suggests that: (i) thermal tolerance tests using resting fish may not provide accurate information on the vulnerability of many fish species to rising temperatures and/or heat waves and (ii) that researchers should use more ecologically relevant protocols that incorporate appropriate levels of activity for the species of interest. For example, Nelson *et al.* (2024) recently showed that Arctic char (*Salvelinus alpinus*) choose to swim rather than rest when exposed to increasing temperatures during a CT_Max_ test. However, the CTS_Max_ protocol is unlikely to represent a swimming challenge experienced by many fishes in nature. Thus, it is incumbent upon researchers to design protocols that mimic swimming behaviours and speeds of the species of interest in nature if their goal is to understand how climate-driven changes in temperature may impact a species’ resilience or vulnerability, and guide conservation strategies. Preferably gathering such data before experiments are performed. To our knowledge, only two studies have directly examined how low to moderate swimming speeds affect fish thermal tolerance, and the data is contradictory. Fish and Nelson (2013) reported that striped bass (*Morone saxatilis*) swimming at 50% of their estimated U_crit_ had a higher average CT_Max_ (~37.8 vs. 37.2°C) than when measured at rest, whereas no effect of swimming at ~0.8 body lengths s^−1^ on CT_Max_ was reported by Nelson *et al.* (2021) for black nose dace (*Rhinichthys atratulus*).

### Incorporating environmental variability

Environmental variability occurs over multiple temporal scales (e.g. tidal, diurnal, seasonal etc.), and the magnitude and frequency of these variations affect the nature and scale of an organism’s physiological response to changes in temperature. This suggests that measures of thermal tolerance need to be performed on fish that are exposed to temperature changes observed in nature (or in culture) prior to testing, and that the test incorporates changes in other environmental parameters that co-vary with temperature. Indeed, lab-based acclimation protocols designed to mirror the variable temperatures that ectotherms experience in the wild are being used increasingly in the evaluation of fish thermal tolerance (Kraskura *et al.,* 2024; Morash, 2024; Waterbury *et al.,* 2024). This is encouraging. Interestingly, however, there is little evidence that fluctuating temperature regimes that mimic natural diurnal temperature changes result in significantly altered CT_Max_ values as compared with static temperature acclimation (i.e. daily mean temperature appears to be the most important determinant of CT_Max_) (Kraskura *et al.,* 2024; Morash, 2024). Furthermore, although hyperoxia occurs in many shallow aquatic environments during the day (e.g. estuaries, tide pools, mangroves, coral reefs), and has been shown to increase the metabolic capacity of fishes (i.e. MMR and AS), it has little to no (≤0.8°C) effect on CT_Max_ even when this test is performed at ecologically relevant heating rates (McArley *et al.,* 2021; Kuchenmüller *et al.,* 2024; Sandrelli *et al.,* 2024; Raby *et al.,* 2025b; Silva-Garay *et al.,* 2025). Collectively, these data suggest that while cycling temperatures and hyperoxia can have important implications for fitness related traits (e.g. growth, reproduction, digestion, predator avoidance, swimming performance) at high temperatures that have important ecological implications (Morash *et al.,* 2018; Kuchenmüller *et al.,* 2024; Morash, 2024), it is not critical that they be incorporated into CT_Max_ protocols that assess upper thermal (i.e. lethal) tolerances.

One of the other major abiotic challenges associated with climate change is hypoxia (low environmental oxygen levels), and this phenomenon often co-occurs with increases in temperature in coastal areas. For example, at aquaculture cage-sites (Stehfest *et al.,* 2017; Burke *et al.,* 2020), in estuaries, as well as in shallow lakes and ponds (Smale *et al.,* 2019; Sampiano *et al.*, 2021; Whitney, 2022; Shinohara *et al.,* 2023). Several studies have evaluated the effects of acute and long-term (acclimation) exposure to hypoxia on the *in vivo* thermal tolerance (CT_Max_) of fishes. Collectively, these studies provide several important insights. First, acute hypoxia only results in a reduction in CT_Max_ when the decrease in water O_2_ content is severe (i.e. <50–60% air saturation) and well below the level of hypoxia where aerobic scope is significantly diminished (Ellis *et al.,* 2013; Ern *et al.,* 2016 and 2017; Gamperl *et al.,* 2025). These studies have important implications for the relevance of the Oxygen and Capacity Limited Thermal Tolerance (OCLTT) paradigm (Pörtner *et al.,* 2017) to the acute thermal tolerance of fishes, and importantly, Ern *et al.* (2016) provide a useful metric that can be used to assess how upper temperature tolerance (CT_Max_) is affected by acute hypoxia. This metric is the PCT_Max_, the threshold dissolved oxygen (DO) level below which CT_Max_ decreases proportionally with further decreases in DO. Second, whether chronic hypoxia exposure improves the CT_Max_ of fishes (i.e. affords cross-tolerance to another oxygen-limited challenge) depends on the water O_2_ level at which the test is conducted. For example, neither Motyka *et al.* (2017), Del Rio *et al.* (2019) nor Reemeyer and Chapman (2024) were able to demonstrate that acclimation to hypoxia improved the CT_Max_ of fishes when measured under normoxia. In contrast, Leeuwis *et al*. (2021), McDonnell *et al.* (2021) and Reemeyer and Chapman (2024) (although the difference was not quite significant), reported that acclimation to severe hypoxia improved the thermal tolerance of fishes at low oxygen levels. That is, cross-tolerance only occurs when fish are tested at oxygen limiting conditions of a similar magnitude to that experienced during acclimation.

### Pathogen infection and thermal tolerance

Finally, it is becoming clear that pathogen load can be very important in determining whether fish succumb to periods of high temperature. For example, Burke *et al.* (2020) concluded that high temperatures and moderate hypoxia (~70% air saturation) were the primary cause of the 2.9 million salmon deaths that occurred in sea-cages in Newfoundland in the summer of 2019. However, more recent data on the effects of these conditions on fish behaviour and physiology (Beemelmanns *et al.,* 2020; Gamperl *et al.,* 2020 and 2021, Zanuzzo *et al.,* 2020; Beemelmanns *et al.,* 2021a, 2021b) do not support this assertion. The salmon in these cages did not experience temperatures >19.5°C (Gamperl *et al.,* 2021). Large (>800 g) salmon from similar stocks tolerate temperatures as high as 21°C for several weeks when also exposed to water O_2_ levels of 60–70% saturation (lower than reported in the cages) without mortalities (Gamperl *et al.,* 2020). Finally, the critical oxygen tension/limiting oxygen saturation (LOS) for Atlantic salmon is ~63–67% at 22°C (Barnes *et al*., 2011; Remen *et al.,* 2013), considerably below the water oxygen level reported in the cages by Burke *et al.* (2020). Thus, it is clear that there were other complicating factors. For example, the salmon were being treated for sea lice (Burke *et al.,* 2020) and this was likely a critical/key factor. Overton *et al.* (2019) report that sea lice treatments at cooler temperatures can result in salmon mortalities ranging from 15 to 30%. In addition, Godwin *et al.* (2020) showed that this species’ thermal tolerance depends on lice load, and that ‘high’ levels of lice infestation decreased the survival of post-smolt salmon at 19°C by 25%. The interpretation that the temperature-dependent mortality of fishes at high temperatures may result from temperature × pathogen interactions is also supported by recent data for several fishes (but see Powell & Gamperl, 2016). For example, a relationship between increasing water temperature and amoebic gill disease (AGD) prevalence has been reported in Atlantic salmon cultured in several countries (Benedicenti *et al.,* 2019). Bonville *et al.* (2024) found that the severity of pumpkinseed sunfish (*Lepomis gibbosus*) infection with trematode parasites was negatively related to CT_Max_, and that fish infected with yellow grub parasites showed decreased survival in the days following CT_Max_ tests. Genin *et al.* (2020) suggest that while heat waves (‘warming events’) may not directly kill coral reef fish, bacterial infections that occur following these events may result in mass mortality. Finally, a meta-analysis by Tomamichel *et al.* (2025) showed that high temperatures increase parasite-induced host mortality. These studies strongly suggest that: (i) researchers ensure that their fish are largely pathogen free if their assessment of thermal tolerance is with regard to ‘healthy’ fish, (ii) if increased pathogen prevalence is associated with high temperatures in the wild or in culture, that lab-based studies of thermal tolerance need to incorporate appropriate pathogen loads and (iii) if using wild fish, it would be beneficial to estimate the extent to which the fish are dealing with parasites and/or other infections. Additionally, the above studies make it clear that we need more research that examines the mortality of fishes for a prolonged period following exposure to temperatures at or near a species’ thermal tolerance.

## Long-term changes in temperature and thermal tolerance

To this point, we have mostly addressed the use of measures of thermal tolerance with regard to short-term (acute) fluctuations in temperature. Nonetheless, average global water temperatures are increasing, and this will likely exacerbate the impact of seasonal changes in temperature (which can be upwards of 30°C in temperate latitudes) on fish survival, ecology and distribution.

### Incremental thermal maximum and minimum tests

To date, only two protocols have been developed to assess the potential impact of seasonal temperature changes, i.e. peak summer and the lowest winter temperatures on fish thermal tolerance. In the incremental maximum and minimum tests (IT_Max_ and IT_Min_ tests; Zanuzzo *et al.,* 2019 and Vadboncoeur *et al.,* 2023, respectively) temperatures are increased or decreased at a steady rate based on changes measured in the natural environment. This method has only been used to measure the upper thermal tolerance of Atlantic cod (*Gadus morhua*) and Atlantic salmon (Zanuzzo *et al.,* 2019; Bartlett *et al.,* 2022; Ignatz *et al.,* 2023; Benfey *et al.,* 2024). However, this research has provided several important findings. A fish’s CT_Max_ can be as much as 4°C higher than their IT_Max_, even when CT_Max_ experiments are performed at ecologically relevant heating rates (2°C h^−1^). CT_Max_ and IT_Max_ are not correlated, and there is no general relation between these two metrics. Parameters that relate to one of these metrics (e.g. heart size) can have the opposite relationship with the other. Finally, CT_Max_ is not, or only poorly, correlated with important life history traits, including growth and swimming capacity (see Table 2 in Morash, 2024). Thus, the quicker (more easily performed) CT_Max_ test should not be used to evaluate the tolerance of fishes to gradually rising temperatures (i.e. over weeks to months). In addition, a ITD_Max_ test was recently used to compare the thermal tolerance of coho salmon (*Oncorhynchus kisutch*) populations in British Columbia (Canada), and to see if this test provided different values for thermal tolerance as compared with a CT_Max_ test (heating rate 18°C h^−1^) (Iacarella *et al.,* 2025a). The ITD_Max_ test, where temperature fluctuated by 3°C and mean temperature was increased by 0.3°C every day, resulted in thermal maxima values that were 2–3°C lower vs. the CT_Max_ test. Furthermore, these authors showed that ITD_Max_ values were not influenced by acclimation temperatures of 14 vs. 18°C, which they attributed to acclimation during the trials. Collectively, these data substantiate the concerns expressed by Rezende *et al.* (2014) that critical thermal limits do not accurately represent the longer-term temperature exposure that is more likely in the environment, and that tolerance landscapes provide a more suitable framework to study temperature tolerance and its potential impact in ecological settings.

One major drawback of the above tests is that their end point is when the fish show signs of ill health (i.e. erratic swimming or trouble maintaining equilibrium) or becomes moribund (i.e. appear to be dying). Thus, conducting these experiments raises ethical concerns, even if the tanks are checked every few hours 24 h a day near the end of the experiment, and questions whether other measures of thermal tolerance should be used. In Zanuzzo *et al.* (2019) and Gamperl *et al.* (2020), the first sign of temperature-dependent effects on their biology was a decrease in, and then the cessation of feeding, the latter occurring at a temperature approximately 5°C lower than their CT_Max_. With regards to biomarkers of sub-lethal stress when exposed to incrementally increasing temperatures, plasma cortisol levels and stress-related gene (transcript) expression have both been examined in these two species. However, they do not predict the onset of sub-lethal stress when these species are exposed to gradually rising (1°C every 5–7 days) temperatures. In the cod, cortisol levels were elevated significantly (by 2.9-fold) at 16°C but returned to control (10°C) levels thereafter (Pérez-Casanova *et al.,* 2008). In the salmon, these values showed a high degree of individual variability and no relation with temperature (Gamperl *et al.,* 2020). Furthermore, liver *hsp70*, *hsp90* and *hsp47* (*serpinh1*) transcript expression levels were significantly higher in salmon by 18°C, the upper boundary for optimal temperatures for this species (Beemelmanns *et al.,* 2021b) and much lower than their CT_Max_.

Very little work has been done regarding appropriate metrics for sub-lethal tolerance when fish are exposed to slowly decreasing temperatures. Nonetheless, Vadboncoeur *et al.* (2023) showed that feeding began to decrease in 8°C-acclimated salmon by 6°C and had stopped by 1°C. In addition, they reported that the liver’s transcript expression of various *hsp70* and 90 paralogues were elevated by 5–6°C, and that plasma cortisol levels were first increased at 2°C. Thus, it is possible that HSP transcript and/or protein expression and plasma cortisol levels may be suitable indicators of sub-lethal low temperature stress in some fishes, respectively. Clearly, more work needs to be conducted on biomarkers of sub-lethal stress and thermal tolerance in these and other fish species when exposed to seasonal changes in temperature.

### The evolution of thermal tolerance

Climate change effects on yearly average water temperatures are projected to proceed at rates between ~0.025 and 0.35°C per decade depending on if, and to what extent, global greenhouse gas emissions are curtailed (IPCC, 2023). Thus, multigenerational experiments are amongst the most vital to perform with regard to predicting whether species will be resilient or vulnerable (i.e. can they adapt?) to climate change-related increases in temperature. Unfortunately, there are only a few studies that have looked at the multi-generational effects of high temperatures on fish thermal tolerance (CT_Max_), and the outlook is not overly positive. Exposing medaka (*Oryzias latipes*) to 30 vs. 20°C for seven years did not result in any detectable change in CT_Max_ (Vallin *et al.,* 2025). Meffe *et al*. (1995) showed that eastern mosquitofish (*Gambusia holbrooki*) held in an artificially heated pond for 60–90 generations only had a CT_Max_ that was ~0.5°C higher than fish from a pond with a natural thermal regime (i.e. ≤0.009°C per generation). Morgan *et al.* (2020) did show that after 6 generations of temperature selection the upper thermal tolerance of zebrafish (*Zebra danio*) increased by an average of 0.04°C per generation, and Andreassen *et al.* (2025) reported that increased upper thermal tolerance after seven generations was concomitant with enhanced cold tolerance (CT_Min_; i.e. an increased tolerance to temperature extremes). However, Morgan *et al.* (2020) also concluded that given the rate at which global temperatures are increasing, and that it appears that improvements in upper thermal tolerance in this species reach a ‘hard ceiling’, that there is a low potential for ‘evolutionary rescue’ in tropical fish from climate change. These results are also concordant with Sandblom *et al.* (2016) who reported that while the CT_Max_ of European perch (*Perca fluviatilis, L.*) exposed to long-term increases in environmental temperature (5–10°C) for 30 years was elevated by 2.2°C relative to reference fish, the difference between normal environmental temperatures and CT_Max_ (i.e. this species’ thermal safety margin) decreased from 10.1 to 4.6°C. Finally, these results agree with the analyses performed by Molina *et al.* (2024) which concluded that while plasticity can partly ameliorate the effects of global warming in the long_term, it will not be able to fully compensate for continually rising temperatures.

The above experiments were conducted on a limited number of species and only assessed the upper thermal tolerance of fishes using CT_Max_ protocols that employed heating rates ≥18°C h^−1^. Thus, similar experiments need to be conducted on other species, and using protocols (e.g. IT_Max_ or TD_Max_ tests) that provide fishes the opportunity to utilize inherent physiological plasticity, before we can effectively assess whether: evolutionary adaptation will allow species to survive temperature increases over the next few decades; or require that they seek out waters with different temperature characteristics (i.e. alter their latitudinal or depth distribution). However, there are some data on the hereditability of thermal tolerance that provide insights into whether fish populations may adapt to climate change. Charo-Karisa *et al.* (2005) and Ma *et al.* (2007) report that the heritability of cold tolerance in tilapia (*Oreochromis niloticus*) and red drum (*Sciaenops ocellatus*) is low to moderate (0.09 and 0.32 ± 0.12, respectively). In addition, Benfey *et al.* (2024), Gonen *et al.* (2024) and Ignatz *et al.* (2025) performed CT_Max_ and IT_max_ experiments on Atlantic salmon using large family-based experiments, and concluded that these metrics of thermal tolerance were only moderately hereditable (i.e. values were 0.47 and 0.40 in Benfey *et al.,* 2024, 0.2 and 0.25 in Ignatz *et al.,* 2025, and 0.16 for IT_Max_ in Gonen *et al.,* 2024). The latter two experiments used ecologically relevant heating rates. Thus, like the experiments of Frederik Jutfelt’s group (Morgan *et al.,* 2020), these data suggest that selection of fish for thermal tolerance may not keep pace with climate change. However, all studies on Atlantic salmon reported very large inter-family and inter-individual variation in these metrics of thermal tolerance, and this implies that there may be enough genetic diversity already in these highly polygenic traits to allow for this species to better resist future climate warming. For example, mean family values for IT_Max_ in Benfey *et al.* (2024) ranged from 21.9 to 25.3°C (overall mean of 23.2°C), and the most tolerant fish had an IT_Max_ of 27.4°C. Nonetheless, salmonids, cyprinids and suckers are (pseudo-) tetraploid, and many sturgeon are polyploid, unlike most fishes, which are diploid. Thus, experiments on these former species may overestimate the genetic potential for many fishes to adapt to climate change or other stressors.

Obviously, multi-generational experiments will be difficult on species that take years to become reproductively mature and would require substantial long-term funding. However, there may be opportunities to collaborate with industries such as those with warm thermal effluents that are discharged into the environment (i.e. nuclear power plants; Meffe *et al.,* 1995; Sandblom *et al.,* 2016: or AI data centres), or the aquaculture sector, on such questions. For example, several Atlantic salmon companies now incorporate thermal tolerance into their selective breeding programs, and this could provide fish for more in-depth experiments on the evolution of thermal tolerance.

## Summary and concluding remarks

To predict the potential impacts of climate change-related environmental challenges on fish populations and implement conservation and management strategies to mitigate them, it is critical that we have accurate data on their sub-lethal and lethal tolerances and temperature-related ‘safety margins’. Several tests are currently being used for this purpose. Below, we provide several recommendations (see the ‘Text Box’). Our goal is to make measures of thermal tolerance more ecologically relevant and better able to predict the impacts of climate change on fish populations.

Box 1.
**Recommendations to ensure that temperature tolerance experiments generate data that are ecologically and climate change-relevant.**

1. Researchers need to be more precise and explicit regarding connections of their research to climate change.
2. If doing experiments that assess acute upper thermal tolerance, focus on life stages that are likely to be exposed to (most vulnerable to) such temperature changes. If unknown, assess the susceptibility of multiple stages to temperature effects.
3. Carefully consider the experimental conditions that fish are exposed to before conducting tests of thermal tolerance (e.g., acclimation temperature and duration of acclimation, constant vs. fluctuating temperatures, oxygen levels, salinity, feeding regimen, swimming activity etc.), and report all relevant details as part of publications.
4. Avoid using UILT and LILT tests, and heating rates > 4^o^C h^-1^ in CT_Max_, tests because they yield inaccurate estimates of thermal tolerance and safety margins. If using a test that involves gradual warming, use a rate appropriate for that species based on its ecology. We recommend rates of heating for CT_Max_/CT_Min_ tests and IT_Max_/_Min_ and TD_Max_ studies of 1-2^o^C h^-1^ and 0.2 - 5^o^C week^-l^ (depending on latitude), respectively to facilitate comparisons between species or life stages.
5. Whenever possible, measure temperature at peak heart rate (T_peak_) or temperature that arrhythmias begin at (T_arr_) on free-swimming fish rather than using the F_H_,_Max_ protocol.
6. Investigate the impact of heat waves on fishes using IT_Max_ or TD_Max_ tests, or using protocols similar to McMahon *et al.* (2024). Further, fish should be monitored after temperatures are returned to pre-heat wave or IT_Max_ conditions for an extended period.
7. More studies need to focus on sublethal effects of temperature changes because they may be more prescriptive than CT_Max_, IT_Max_ etc. This recommendation agrees with the meta-analysis of Molina *et al.* (2025) which reports ‘conclusive evidence’ that so-called thermal safety margins based on ‘critical temperature’ values greatly overestimate resilience to warming. For example, consider using behavioural metrics of sub-lethal thermal tolerance (e.g. activity, agitation, loss of appetite, cessation of feeding), as changes in behaviour can provide ecologically-relevant information on thermal tolerance.
8. Incorporate other concurrent variables if a species is likely to encounter more than one challenge in conjunction with high or low temperatures. For example, hypoxia, pathogen load, changes in salinity, swimming activity etc.
9. Consider looking at the temperature-dependent biology of major prey or predators because they may lead to population declines or changes in the distribution of the fish species being studied.
10. Studies of temperature-related performance or tolerance in fish that spend considerable time swimming should use prolonged swimming speeds in their therml tolerance tests that match those recorded in nature.
11. We advocate for a focus on ecologically-relevant comprehensive experiments (including multigenerational studies and those that focus on mechanisms determining tolerance) to better predict how climate change will impact fish biology and survival.

We suggest that some tests should be discontinued (e.g. ILT tests, CT_Max_ tests at heating rates > 4°C h^−1^; 0.067°C min^−1^) if the goal is to estimate thermal tolerance of fishes in the wild or in aquaculture. Furthermore, it is not possible at present to estimate values of sub-lethal acute thermal tolerance using transcript or protein expression due to the scarcity of data in this area, and the apparent temperature-dependent time lag between the induction of gene expression and protein synthesis. Rather, if ‘high-throughput’ assessments of the CT_Max_ of fishes are required, we suggest that researchers perform CT_Max_ experiments at ecologically relevant heating rates on groups of fish at the same time and sample them as they succumb (Zanuzzo *et al.,* 2019).

Clearly, the ‘Rapid Screening Protocol’/F_H,Max_ test, while providing valuable information on the F_H_ response and cardiac limits of fishes when exposed to acute temperature changes, does not provide quantitatively precise information on their acute thermal tolerance. Instead, it is recommended that alternative approaches using free-swimming animals be conducted (e.g. using the measurement of bioelectric potentials, biologging or telemetry, or *in vivo* blood flow) that allow for more accurate assessments of thermal tolerance, as well as physiological and behavioural data to be simultaneously recorded. Finally, it is not recommended that CT_Max_ tests (even at ecologically relevant rates of heating) be used to estimate the tolerance of fishes to prolonged changes in temperature. CT_Max_ does not predict the tolerance of fishes to long-term (over weeks to months) temperature changes (e.g. as assessed by the IT_Max_ or TD_Max_ tests), and the evidence to date indicates that the primary biological processes that determine these two metrics of thermal tolerance are not the same.

Finally, we have two appeals. First, that ecologists, population modellers, and those involved in fish management and conservation not take reported values of a species’ thermal tolerance in the literature at ‘face value’, but endeavour to understand what various estimates of thermal tolerance actually mean, their implications, and whether (and how) they can be incorporated into determining tolerance limits and safety margins with respect to protecting and conserving fish populations and biodiversity. Second, we implore fish biologists to: (i) think carefully about the relevance of the question they are asking and the best way to address it experimentally. Loss of equilibrium (or becoming moribund in long-term experiments) may not be the best (most relevant) measure of thermal tolerance with regard to ecological implications for wild fish or effects on cultured fishes. For certain species, under certain circumstances, sub-lethal measures of thermal tolerance may be more insightful. For example, agitation temperature may be a more appropriate measure of acute thermal tolerance (see Kochhann *et al.,* 2021), and loss of appetite and cessation of feeding may be more relevant measures of long-term thermal limits than IT_Max_ (see Pérez-Casanova *et al.,* 2008; Gamperl *et al.,* 2020)?. (ii) Not shy away from comprehensive (including multi-stressor, multi-life stage and multi-generational) and ecologically relevant (i.e. based on the rate/temporal nature of temperature changes, and an appropriate level of fish activity) experiments. (iii) Incorporate aspects into their studies on mechanisms or behaviours that influence/define fish sub-lethal and lethal temperature tolerances and/or may determine their vulnerability to climate change**.** Such experiments take larger financial and time commitments than many of the current ‘high-throughput’ protocols that are being used, but would have several tangible benefits, including training of the next generation of scientists in such experimental approaches. A major challenge to these important fields is exciting the next generation of scientists about mechanistic studies, training them and getting them to make these approaches a central part of their own research.

## Supplementary Material

Web_Material_coag044

## Data Availability

This article does not contain any original data.
