# Supplementary material for: The relevance and application of measures of thermal tolerance to fish ecology, conservation and aquaculture
Source: Conserv Physiol. 2026 Jul 7;14(1):coag044. doi: 10.1093/conphys/coag044 (PMC13339953; doi:10.1093/conphys/coag044)

Gamperl and Stevens: The Relevance and Application of Measures of Thermal Tolerance to Fish Ecology, Conservation and Aquaculture

Common Name	Species	Tpeak	CT_{Max}	% Diff	Diff (°C)	Estimated CT_{Max}	Diff (Real vs. Estimated) °C
coho salmon	*Oncorhynchus kisutch*	21.3	27	26.76056	5.7	27.09431	0.09431
rainbow trout	*Oncorhynchus mykiss*	23.7	29	22.36287	5.3	29.30399	0.30399
redband trout	*Oncorhynchus mykiss*	23.8	29.8	25.21008	6	29.39606	-0.40394
redband trout	*Oncorhynchus mykiss*	23.5	29.7	26.38298	6.2	29.11985	-0.58015
redband trout	*Oncorhynchus mykiss*	23.2	29.3	26.2931	6.1	28.84364	-0.45636
redband trout	*Oncorhynchus mykiss*	24.2	28.8	19.00826	4.6	29.76434	0.96434
brook char	*Salvelinus fontinalis*	26.1	29.8	14.17625	3.7	31.51367	1.71367
Arctic Cod	*Boreogadus saida*	7.8	14.9	91.02564	7.1	14.66486	-0.23514
Arctic Cod	*Boreogadus saida*	12.3	15.5	26.01626	3.2	18.80801	3.30801
Arctic Cod	*Boreogadus saida*	10.9	17.1	56.88073	6.2	17.51903	0.41903
Arctic char	*Salvelinus alpinus*	16.1	24.5	52.17391	8.4	22.30667	-2.19333
Arctic char	*Salvelinus alpinus*	18.1	25.2	39.22652	7.1	24.14807	-1.05193
Arctic char	*Salvelinus alpinus*	20.7	26.4	27.53623	5.7	26.54189	0.14189
Arctic char	*Salvelinus alpinus*	24.1	27.2	12.86307	3.1	29.67227	2.47227
opaleye	*Girella nigricans*	25.7	31.2	21.40078	5.5	31.14539	-0.05461
opaleye	*Girella nigricans*	26.4	31.3	18.56061	4.9	31.78988	0.48988
opaleye	*Girella nigricans*	30.4	35	15.13158	4.6	35.47268	0.47268
opaleye	*Girella nigricans*	29.5	35	18.64407	5.5	34.64405	-0.35595
chinook salmon	*Oncorhynchus tshawytscha*	21.2	26.5	25	5.3	27.00224	0.50224
pearl danio	*Danio albolineatus*	33.3	38.9	16.81682	5.6	38.14271	-0.75729
glowlight danio	*Danio choprae*	31.1	37.2	19.61415	6.1	36.11717	-1.08283
zebrafish	*Danio rerio*	33.6	39.9	18.75	6.3	38.41892	-1.48108
rainbow trout	*Oncorhynchus mykiss*	25	29	16	4	30.5009	1.5009
rainbow trout	*Oncorhynchus mykiss*	25	30	20	5	30.5009	0.5009
rainbow trout	*Oncorhynchus mykiss*	25.5	30.4	19.21569	4.9	30.96125	0.56125
rainbow trout	*Oncorhynchus mykiss*	26.8	30.7	14.55224	3.9	32.15816	1.45816
rainbow trout	*Oncorhynchus mykiss*	27.6	31.1	12.68116	3.5	32.89472	1.79472
rainbow trout	*Oncorhynchus mykiss*	26.2	31.2	19.08397	5	31.60574	0.40574
rainbow trout	*Oncorhynchus mykiss*	20	29	45	9	25.8974	-3.1026
sea bass	*Dicentrarchus labrax*	21.8	31.3	43.57798	9.5	27.55466	-3.74534
Atlantic salmon	*Salmo salar*	20.2	27.7	37.12871	7.5	26.08154	-1.61846
	**Averages**	23.39032	29.01935	27.32497	5.629032	29.01887	-0.000484839
				**Max**	9.5	38.41892	3.30801
				**Min**	3.1	14.66486	-3.74534

Common Name	Species	Tarr	CT_{Max}	% Diff	Diff (°C)	Estimated CT_{Max}	Diff (Real vs. Estimated) °C
coho salmon	*Oncorhynchus kisutch*	22	27	22.72727	5	25.336	-1.664
rainbow trout	*Oncorhynchus mykiss*	25.1	29	15.53785	3.9	28.22365	-0.77635
redband trout	*Oncorhynchus mykiss*	24.9	29.8	19.67871	4.9	28.03735	-1.76265
redband trout	*Oncorhynchus mykiss*	23.8	29.7	24.78992	5.9	27.0127	-2.6873
redband trout	*Oncorhynchus mykiss*	24.5	29.3	19.59184	4.8	27.66475	-1.63525
redband trout	*Oncorhynchus mykiss*	25.1	28.8	14.74104	3.7	28.22365	-0.57635
brook char	*Salvelinus fontinalis*	27.4	29.8	8.759124	2.4	30.3661	0.5661
Arctic Cod	*Boreogadus saida*	13.4	15.5	15.67164	2.1	17.3251	1.8251
Arctic Cod	*Boreogadus saida*	11.1	17.1	54.05405	6	15.18265	-1.91735
Arctic char	*Salvelinus alpinus*	19.1	24.5	28.27225	5.4	22.63465	-1.86535
Arctic char	*Salvelinus alpinus*	22.4	25.2	12.5	2.8	25.7086	0.5086
Arctic char	*Salvelinus alpinus*	23.4	26.4	12.82051	3	26.6401	0.2401
Arctic char	*Salvelinus alpinus*	26.2	27.2	3.816794	1	29.2483	2.0483
opaleye	*Girella nigricans*	27.5	31.2	13.45455	3.7	30.45925	-0.74075
opaleye	*Girella nigricans*	28.6	31.3	9.440559	2.7	31.4839	0.1839
opaleye	*Girella nigricans*	31.8	35	10.06289	3.2	34.4647	-0.5353
opaleye	*Girella nigricans*	31.2	35	12.17949	3.8	33.9058	-1.0942
chinook salmon	*Oncorhynchus tshawytscha*	22.4	26.5	18.30357	4.1	25.7086	-0.7914
pearl danio	*Danio albolineatus*	36.9	38.9	5.420054	2	39.21535	0.31535
glowlight danio	*Danio choprae*	33.2	37.2	12.04819	4	35.7688	-1.4312
zebrafish	*Danio rerio*	36.6	39.9	9.016393	3.3	38.9359	-0.9641
rainbow trout	*Oncorhynchus mykiss*	25.7	29	12.84047	3.3	28.78255	-0.21745
rainbow trout	*Oncorhynchus mykiss*	25.9	30	15.83012	4.1	28.96885	-1.03115
rainbow trout	*Oncorhynchus mykiss*	27.7	30.4	9.747292	2.7	30.64555	0.24555
rainbow trout	*Oncorhynchus mykiss*	27.6	30.7	11.23188	3.1	30.5524	-0.1476
rainbow trout	*Oncorhynchus mykiss*	29.1	31.1	6.872852	2	31.94965	0.84965
rainbow trout	*Oncorhynchus mykiss*	27.2	31.2	14.70588	4	30.1798	-1.0202
rainbow trout	*Oncorhynchus mykiss*	20.3	29	42.85714	8.7	23.75245	-5.24755
sea bass	*Dicentrarchus labrax*	25.6	31.3	22.26563	5.7	28.6894	-2.6106
sockeye salmon	*Oncorhynchus nerka*	24.1	25.9	7.46888	1.8	27.29215	1.39215
sockeye salmon	*Oncorhynchus nerka*	24	24.3	1.25	0.3	27.199	2.899
sockeye salmon	*Oncorhynchus nerka*	24.2	24.4	0.826446	0.2	27.3853	2.9853
sockeye salmon	*Oncorhynchus nerka*	23	25.6	11.30435	2.6	26.2675	0.6675
sockeye salmon	*Oncorhynchus nerka*	23.2	24.1	3.87931	0.9	26.4538	2.3538
sockeye salmon	*Oncorhynchus nerka*	24.4	23	-5.7377	-1.4	27.5716	4.5716
sockeye salmon	*Oncorhynchus nerka*	23.1	23.5	1.731602	0.4	26.36065	2.86065
sockeye salmon	*Oncorhynchus nerka*	24.5	23.4	-4.4898	-1.1	27.66475	4.26475
	**Averages**	25.3027	28.41081	13.39111	3.108108	28.41246757	0.001656757
				**Max**	8.7	39.21535	4.5716
				**Min**	-1.4	15.18265	-5.24755

A

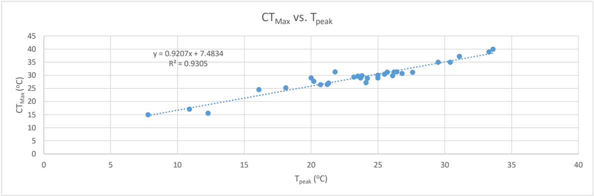

B

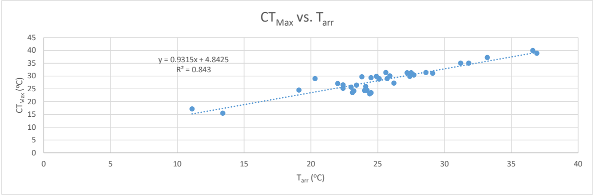

C

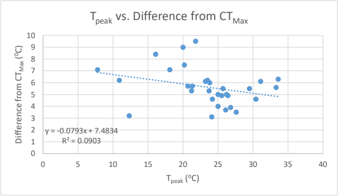

D

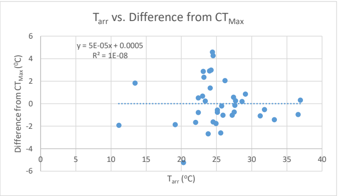

E

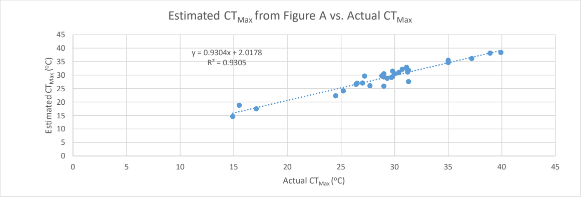

F

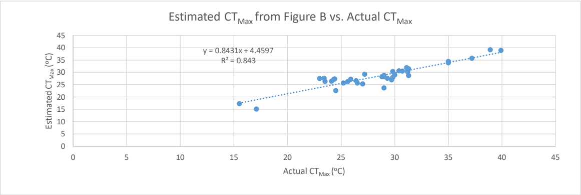

G

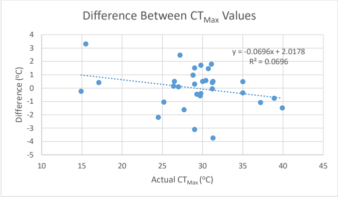

H

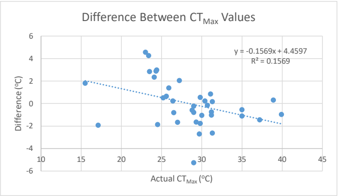

Supplement: Web_Material_coag044 [file web_material_coag044.zip › Supplemental File_Submitted_coag044.pdf]
